# The Impact of Health Human Resources Policies in Primary Care Nursing: A Qualitative Study

**DOI:** 10.3390/ijerph16193653

**Published:** 2019-09-28

**Authors:** María Madrazo-Pérez, Paula Parás-Bravo, Esperanza Rayón-Valpuesta, Cristina Blanco-Fraile, Domingo Palacios-Ceña

**Affiliations:** 1 Faculty of Nursing, University of Cantabria; Avda Valdecilla s/n. C.P., 39008 Cantabria, Spain; maria.madrazo@unican.es (M.M.-P.); cristina.blanco@unican.es (C.B.-F.); 2 Researh Nursing Group IDIVAL; Calle Cardenal Herrera Oria s/n. C.P., 3901 Cantabria, Spain; 3 Department of Nursing, University of Complutense; Plaza de Ramón y Cajal, 3, C.P., 28040 Madrid, Spain; esperanza.rayon@unavarra.es; 4 Department of Physiotherapy, Occupational Therapy, Rehabilitation, and Physical Medicine, University Rey Juan Carlos; Avda de Atenas s/n. C.P., 28922 Madrid, Spain; domingo.palacios@urjc.es; 5 Research Group of Humanities and Qualitative Research in Health Science of Universidad Rey Juan Carlos (Hum&QRinHS), Universidad Rey Juan Carlos, 28922 Madrid, Spain

**Keywords:** organization and administration, organizational culture, primary care nursing, personnel selection, nurses, qualitative research

## Abstract

Background: Organizational culture plays a key role regarding organizational outcomes and determining strategies, goals, and modes of operating which is associated with higher rates of worker morale, turnover and lower adverse events related to patient quality of care issues. Aim: to describe the impact of the relocation of nurses from hospitals and other contexts to primary care from the perspective of primary care nurses. Methods: A qualitative, focused ethnographic study. Site: Cantabro Health Service, Cantabria, Spain. Purposeful sampling methods were used to include nurses who were working in primary care during the study, and who had previous experience of at least one year in primary care. Observation (385 hours, 7 months) and in-depth interviews (17) were used to collect data. A thematic analysis was applied. Results: Four themes emerged from the data: (a) staff policies applied, (b) beliefs regarding the newly incorporated nursing staff, (c) reasons for relocation to primary care, and (d) concern for the future. Conclusions: In primary care, the relocation of non-qualified nursing professionals who are at the end of their career may have a negative impact on the organizational culture. It is necessary to research the most appropriate measures for guaranteeing a satisfactory work environment based on nurses who are qualified in primary health care settings.

## 1. Introduction

At present, societies must face an evolving dynamic health reality, which forces governments to organize, plan and adapt their health resources to meet new challenges [1]. These new health challenges include chronicity, disability, care for vulnerable populations and for those who are at risk of exclusion [1]. Many of these challenges are related with social aspects that influence the health of the population, such as poverty, unemployment, difficulties concerning the access and distribution of health resources, and marginalization [1]. In Primary Care (PC), health and social aspects converge, which has a joint influence on the individual, the family and the community [1]. According to the WHO, PC “is about caring for people, rather than simply treating specific diseases or conditions and it provides comprehensive, accessible, community-based care that meets the health needs of individuals throughout their life” [2].

Additionally, the model of health management and organization influences the distribution of its resources (material, economic and human), and therefore, this influences the healthcare received [1,3,4]. On the other hand, the management of resources and the application of norms and regulations also affects the organization of health professionals and the planning of interventions and functions within the health system [4]. Within the process of health organization and management, it is necessary to describe and deepen the available knowledge concerning the different organizational cultures that constitute and coexist within the health system [5,6]. The expression of this culture is embedded in the routine and patterns of daily living and inferred from the words, actions, interactions, and emotions of members of the group [5,7]. 

Culture is defined as a pattern of shared basic assumptions that are learnt by a group via solving their problems of external adaptation and internal integration, which has worked well enough to be considered valid and, therefore, to be taught to new members as the correct way to perceive, think, and feel in relation to those problems [5,8]. In addition, culture determines both our individual and collective behavior, ways of perceiving, thought patterns, and values. Moreover, the organizational culture plays a key role regarding organizational outcomes and determining strategies, goals, and modes of operating which is associated with higher rates of worker morale, turnover and lower adverse events related to patient quality of care issues [5,9]. However, working on the more profound elements of culture requires reflecting on how corporate beliefs and values surface and manifest among health care professionals with regards work practice, group dynamics, and leadership as well as influencing expected and actual behavior [5]. Insight into the dynamics of culture provides a deeper understanding not only of why various groups of people or organizations can be so different, but also why it is so hard to change them. Even more importantly, if we understand culture better, we will understand the forces acting within us that define who we are, and which reflect the groups that we identify with and to which we want to belong [5,8].

These organizational cultures appear in health care and nursing professionals [6,10,11], and they determine their manner of caring for and applying health interventions [10,11], their integration into the team with autonomy and their own role [3,5,12], as well as their way of perceiving ill people, the family and the community [6]. Within the organizational culture, it is necessary for the members of the group to share and accept norms, values, beliefs in their role and identity within this context [6,11]. The American Academy of Nursing on Policy [1] reported that values and scope of practice in PC nursing must possess specific and specialized knowledge and skills for PC, must facilitate access to the PC population, contribute towards reducing the inequalities and include populations at risk (ethnic and racial minorities). Nursing interventions are holistic and family-centered, and nurses must work in collaboration with physicians and other members of the health workforce to optimize patient care and health. 

A challenge that these organizational cultures must face is incorporating new members that do not share, accept or know the culture [3,5], provoking conflicts within the group [5]. In PC, the incorporation, recruitment and retention of nurses is influenced by factors such as role and professional autonomy of nurses [13,14,15], work satisfaction [15], low expectation of staff promotion [3,15], absence of specialized training in primary care [16], age and years of experience of nurses [11,17,18], and culture of the shared workplace (values and beliefs of the mission of the staff in the same) [11,15]. 

Lastly, the incorporation of nurses into P, who lack specialized training and have PC work expectations that are based on myths and preconceptions (e.g., work that is less intense compared to the hospital, having a flexible timetable), and replacing veteran nurses in primary care [17], may hamper the integration of new nurses into PC teams and generate loss of skills, experience and intelligence about local communities [18].

No studies to date have investigated the effects of the health organization (human resources) and their interventions on the organizational culture of nursing. In addition, to our knowledge, no qualitative study exists exploring the perspective of primary care nurses regarding the application of norms that condition the incorporation of new nurses in primary care. The following questions guided this study: What are the shared roles and values of PC nurses? What is their experience regarding new nurses that relocate to PC from hospitals? The aims of this study were (a) to describe the impact that the application of the Spanish law on the management of human resources in primary care (BOE Ley 9/2010) [19,20] has on the relocation of nurses from hospitals to PC in the region of Cantabria (Spain) and regarding the shared roles and values of Spanish PC nurses and (b) to describe the perspective of PC nurses regarding the incorporation of new nurses coming from other contexts and/or without specialization in PC.

## 2. Materials and Methods

This study was conducted following the Consolidated Criteria for Reporting Qualitative Research (COREQ) guidelines [21] (Table S1. Coreq Checklist), and the Creswell & Poth recommendations for ethnography studies [22].

### 2.1. Design

A qualitative, focused ethnographic study was conducted [7,22]. These studies are used to develop holistic descriptions and an understanding of practice in health care [7]. Within the health care context, this approach recognizes cultural groups within different settings, such as people who share common settings (health care professionals) or groups of people who share a similar issue (profession or disease). Used in health care, the ethnographic approach can gain an insider perspective on everyday life through the researcher’s engagement with people over time within the cultural group, and using multiple methods of data generation [7,22].

### 2.2. Research Team

Five researchers were involved in this study (five women, one man). All the researchers had experience in qualitative designs. All the researchers held PhDs in health sciences, were University professors, and were not involved in clinical activity. Three members of the research team worked as nurses in PC (MMP, PPB, CBF), although they had no previous relationship with the participants. The positioning of the researchers was established regarding the theoretical framework, their beliefs, prior experience and personal motivations for participating in the research [7] (See Table 1).

### 2.3. Context/Setting

This study was conducted at the ‘Astillero’ Health Clinic (HC), belonging to the Servicio Cantabro de Salud (Cantabria Health Service, Spain). In Spain, the HC represents the basic physical and administration unit via which PC is performed in Spain [26]. The functions of the HC have been regulated since 1984 and they seek to guarantee the right to health by performing integrated activities for health promotion, prevention, assistance and rehabilitation directed both at individuals, considering these in an isolated manner, as well as the social groups and the communities in which these are inserted who, on the other hand, actively participate throughout the entire health process [26]. In the HC featured in this study, the nursing team was formed by professionals who were both specialized and non-specialized in primary care, and who had variable levels of training in community and primary care. This group of nurses have their own organizational culture, as described in Table 2. 

The description of the shared culture of primary care nurses regarding an event (new human resources policy management) is important to describe how this cultural group works and lives via their “emic” perspective [22]. 

In Spain, based on the current regulations [19,20], work relocation of nurses is arranged among different health centers of the public health service, including hospitals, specialty centers, and HCs, encompassing both specialized care (hospital-based) and primary care. These relocations allow nurses to be transferred from hospitals to PC HCs based on the following fundamental criteria: permanent employees, with seniority (based on the number of years worked in nursing). These criteria does not require nurses to be specialized in PC nursing [19,20,27].

### 2.4. Participants

The inclusion criteria consisted of the following: (a) nurses with a university degree in Nursing, (b) whose workplace was PC at the time of the study, (c) with at least one year of experience in PC, (d) able to communicate, and (e), who had signed the informed consent. All participants had to meet the previously defined inclusion criteria. All the professionals who were invited to participate joined the study, with no refusals.

### 2.5. Sampling Strategies

Purposive sampling strategies are well suited to ethnographic studies [7,22]. Purposive sampling can be defined as the selection of potential participants based on specific purposes associated with addressing the research study question or aim [28,29]. Fourteen participants were included within the sample, none of whom withdrew from the study. 

The researchers made initial contact with the patients via the gatekeeper who was the nurse in charge of the health center [7]. The function of the gatekeeper is to facilitate the entrance of researchers into the social and cultural context of the study [7]. The researchers explained the purpose and design of the study to the participants during an initial face-to-face contact session. A one-week period was then granted for participants to decide whether they agreed to participate. During the second face-to-face session, they were asked to provide written informed consent and permission to tape the interviews if they agreed to participate. Following this, data were collected. 

### 2.6. Data Collection

Data were acquired from 1 July 2013 until 30 October 2013. Data collection consisted of a first phase based on unstructured interviews, with open questions such as: how was the incorporation of the new nurses? Do the newly incorporated nurses share the values, beliefs, principles and knowledge of care in primary care? After this phase, areas of interest were identified to determine which items required further examination; therefore, a second phase was necessary based on semi-structured interviews and non-participant observation.

The semi-structured interviews were conducted using an open-ended questions guide based on the unstructured interviews performed in phase one. The question guide it shows in the Table 3.

All the interviews and observations were accompanied by researcher notes. During the interview, the researcher made notes, including a description of the environment, patients’ non-verbal responses to questions, the use of metaphors in patients’ narratives, and other relevant points that emerged in the interview [7].

In total, 55 days of observations (385 hours) were performed, over three months (seven weeks), divided into 55 sessions and with a mean duration of seven hours, focused on the following research areas and interaction moments: (a) welcome/reception session of newly incorporated nurses, (b) nurse training sessions, (c) interdisciplinary team meetings at the HC, (d) meetings of the HC nursing group, (e) sessions of the HC quality commission, (f) clinical sessions involving the interdisciplinary group, and (g) sessions for HC activity coordination and planning.

In total, 17 in-depth interviews took place: five non-structured, and 12 semi-structured interviews (in total 464 minutes), and 17 researcher notes were performed.

Participant recruitment finished when there was repetition in the information obtained from the interviews [7]. This was achieved at the time of the interview with participant no. 14.

### 2.7. Data analysis

First, a complete and literal transcription was drafted for each interview, and observation and researcher field notes were drafted. The texts were then collated for qualitative analysis [30], after which thematic analysis of the data was conducted [7,30]. This process began by pinpointing the most descriptive content to obtain meaningful units. This was followed by a deeper analysis to reduce and identify the most common meaningful group [7,30]. Thus, groups of meaningful units were formed based on similar points or content. This process of thematic analysis was conducted separately for each interview, each observation and each researcher note. Coding and analysis were conducted separately for each interview, each observation and each researcher note on behalf of two researchers (MMP, ERV). Later, the results of the analysis were combined during team meetings, in which the researchers met to discuss the data collection and analysis procedures [7,30]. In case of differences of opinions, the final themes were identified by consensus. QSR NVivo 10 data analysis software was used.

### 2.8. Rigor

The COREQ guidelines [21] were followed. Furthermore, we used criteria by Guba & Lincoln (Table 4) for establishing trustworthiness of the data by reviewing issues concerning data credibility, transferability, dependability and confirmability [7,31,32].

### 2.9. Ethical Considerations

The study was approved by the Cantabria Health Service, based on current legislation and performed in accordance with the Declaration of Helsinki [33]. Informed consent was obtained from all participants included in the study. Data were treated anonymously and confidentially according to the Spanish Personal Data Protection Act [34] and the Biomedical Research Act [35]. 

## 3. Results

In total, 12 women and two men, with a mean age of 47.4 years (SD ± 8.41), participated in this study. The mean work experience in primary care was 12.50 years (SD ± 5.14). Of the nurses who worked in the HC, five came from other primary care HCs (35.71%), five came from hospitals (37.71%), and four came from elsewhere (28.57%).

The themes representing the participants’ perspective were extracted from the non-participant observations, in-depth interviews, and researcher notes. Four themes emerged from the material analyzed: (a) staff policies applied, (b) beliefs regarding the newly incorporated nursing staff, (c) reasons for relocation to primary care, and (d) concern for the future.

The themes presented below were aimed at depicting a holistic cultural portrait, in which researchers described a working set of rules as to how the nurses’ culture-sharing group functions, in the case of the incorporation of new nurses [22]. We included the narratives taken directly from the data collection regarding the themes identified in this study.

Table 5 shows literal excerpts from the interviews.

**Theme** **1.** *Staff policies applied.*

The nurses narrated that while they understood the need for constant change and evolution in the HC, they felt that the changes underway were contradictory because the new nurses were nurses with an extensive career path, however, based in a hospital context, having no previous contact with primary healthcare.

Nurses narrated how these new nurses come from a hospital culture geared towards illness, where technology is of prime importance and where health promotion and illness prevention is not the priority.

Affirmations made by some nurses such as, *“this has been disastrous”* (E03), show that they perceived that this strategy was altogether wrong.

Regarding the HC nursing teams, the nurses described how the policies for assigning new staff on behalf of the management had not been directed at reinforcing or maintaining a balance between the staffing of nurses and the care and interventions performed in the community.

Primary care nurses assigned the responsibility of this poor staffing strategy to the managers who call for the provision of vacancies, without considering the nurses or the real needs and without considering the repercussion this had on care provision within the HC teams. This leads to conflicts between members of the team and the newly hired nurses.

**Theme** **2.** *Beliefs regarding the newly incorporated nursing staff.*

Primary care nurses shared a series of myths and beliefs concerning the new nurses who were relocated from the hospital. These myths and beliefs support a distorted vision of hospital nurses, characterized by comments such as, *the old glories of Valdecilla believe that they have come to the elephants’ graveyard*.

The nurses based these beliefs on the fact that nurses coming from the hospital had a “fixed”, “civil servant”-like relationship and this condition is related with staff who have low motivations and whose expectations equal poor work involvement.

These beliefs are justified by the perception of the complexity of the nursing role in primary care, based on the essence of their work and their professional autonomy.

On the other hand, they referred to the fact that the vision and conception of work and the objectives of hospital nursing and primary care nursing are totally different. These different views are the ones that lead to conflicts.

**Theme** **3.** *Reason for relocation to primary care*

The nurses included in this study who were relocated into primary care from hospitals narrated that despite having a long professional career as nurses in the hospital, this type of relocation was solitary, met with little support and in an abrupt manner. These nurses experienced conflicting emotions after all the years they had worked at the hospital.

The motivation for the relocation from the hospital to PC was moslty due to professional disenchantment with hospital work and the search for better work conditions, although they lacked knowledge regarding PC.

**Theme** **4.** *Concern for the future.*

With the arrival of the newly relocated staff, in addition to the problems affecting the cohesion of the teams, the presence of considerable work instability amongst primary care nurses (there are no stable contracts, most contracts are temporary) led to a major turnover, affecting the ability for nurses to become involved and to establish relationships of trust with patients.

Nurses described that all the individual and collective work performed in order to build an individualized care model that is oriented towards people and the community was ignored by the managers and was contradictory with the main benefit that primary care provokes, which is the continuity of healthcare.

## 4. Discussion

Our results show how policies for the incorporation of new staff affected the organizational culture of nurses in PC. The presence of beliefs regarding the new nurses who came from the hospital and their motivations, were met with great concern for the future work of PC nurses.

The strategy for the provision and relocation of staff used in the Cantabrian Health Service (BOE Ley 9/2010) [19,20] means that professionals select PC for reasons besides professional activity or their level of qualification/specialization in the sector. This means that professionals with a vast experience in hospital care/specialized care decide to transfer their workplace to PC for reasons such as beliefs of a lesser workload or a fixed morning work shift. This exodus of professionals from specialized care could be alleviated with work strategies that are able to encourage nurses to remain in their workplace. These policies have been implemented with success in countries such as the UK, in which flexible work shifts or professional leave has been granted in nurses over the age of 50 [18].

The findings of authors [11] show that the policies for the selection and recruitment of nursing professionals in their primary care workplaces are especially interesting if we seek favorable work environments that translate into a better care quality. In this sense, it determined that factors such as work, age or the work shift were considered as key elements for professionals.

Nonetheless, in addition to the previously commented factors, another author [17] revealed the importance of professional qualification, seeing as this directly affects the cost of medical care, work satisfaction and patient care. This affects the transfer of knowledge and the provision of quality care to patients. 

In our environment, there are no staff policies adapted to the professional in all phases of professional life. This forces older professionals from the hospital context to choose PC as the last destination two or three years before retirement. This generates alterations in the work of the interdisciplinary team due to the high turnover and the non-specialization of the professionals. Moreover, this negatively affects their work satisfaction and level of stress [36], the turnover hampers the involvement of the nurses and the development of relationships and trust. 

This staff policy is becoming removed from the main aim of PC, which is to provide care to the community [26], which, in part, is based on the relationship of trust with professionals, which facilitates the continuity of such care.

The incorporation of new non-qualified professionals is negatively affecting the organizational culture of PC, and this obviously has a direct repercussion on the care of patients.

The most effective mode of care based on interprofessional teams is met with barriers such as the absence of support and scarce relationships with the rest of the team [3]. 

For all these reasons, the wealth of literature over the last decade on the importance of PC implies the need for managers to establish policies for the provision of staff which are coherent with the professional requirements in this sector. 

Developing policies for the provision and relocation of staff based on the level of qualification and experience on PC, creating in hospital care/specialized positions and schedules adapted to the last years of the professional career and promoting PC specialization can be some of the formulas that contribute to reversing the current situation.

Nonetheless, robust and longitudinal research is necessary to determine which aspects are necessary to guarantee that employment in this context is satisfactory and to ensure that it is dominated by nurses who are qualified in this area [37].

A limitation of this study is that the results cannot be generalized due to the qualitative nature of the data. Furthermore, the authors consider that the main strength of this study is that the application of human resource policies may influence the organizational culture of nurses in the complex primary care context.

## 5. Conclusions

The organizational model in healthcare must evolve towards strategies that adapt to the changing needs of the professionals throughout their professional career. The provision of unqualified staff in PC who are at the end of their career may negatively affect the organizational culture.

The elaboration of staffing policies must be coherent with the professional needs of PC, favoring the incorporation of nurses who are qualified in the area and not for reasons related to the work load or the shift.

In the future, it is necessary to perform research to determine which aspects are necessary in order to guarantee a satisfactory work environment with nurses who are qualified in this sector.

## Figures and Tables

**Table 1:** The positioning of the researchers.

Theoretical framework	The researchers based their approach on ethnographic realism, used by cultural anthropologists, via an objective description of the situation by objectively reporting the information learnt from participants on the field.
Beliefs [23,24,25]	There is evidence that the knowledge of an organization’s culture influences its efficacy and the effectiveness of its production. Before granting importance to its existence and understanding its impact on work life, there were already studies which established a tight relationship between the structure of an organization and performance. Also, the growing interest in identifying the complex interaction of forces that takes place is related with the discovery, on behalf of the directors of the great enterprises, of the potential of culture. At present, there is some experimentation with management models that are very distant from the characteristics that historically identified the success of a company with the existence of visionary foundational leaders, giving greater importance to the relationships between teams.
Prior experience with primary health nurse culture	The interest in studying the nursing culture in primary care is justified by having formed part of the group of health professionals who, during the second part of the 1980s, and in the Autonomous Community of Cantabria, had the opportunity of participating in the health system reform, granting a key role to the new model of care that arose from the Alma-Ata Declaration. This is influenced by having perceived the experience as something new, intense and different, a project in which I had an active role. This shaped me as a professional, helping me to perceive a new way of practicing nursing.
Motivation for the research	To describe the impact of policies and laws on the organizational culture of primary care nurses, via their perspective and their interaction with other health professionals, nursing professionals, the family and the community. The scarcity of international qualitative studies on the topic of study and the absence of publications in Spain, supports the need for qualitative research in this field.

**Table 2:** Primary care nursing culture-sharing group.

Primary Care Nursing Culture-Sharing Group
**Roles and values of primary care nursing:**
The individual is not an isolated entity, rather people are integrated within a family context and within a community. The primary care nurse must care for the individual, the family and the community. The primary care nurse must work together with the community, integrating within the same as a health agent and leading programs and activities for health promotion and illness prevention. Nurses must collaborate together with social leaders and the community, orienting care and actions towards their needs. Nurses from the health clinic act valuing the needs of people and apply care based on intervention protocols, health programs and standardized procedures. All care is preceded by a holistic and comprehensive assessment, acting upon the individual, the family and the community.
**Social relations and hierarchy** [22]:
Nurses accept the existence of a hierarchy among nurses determined by the level of experience and seniority within the health clinic, due to the specialized knowledge in primary care and because of the past experience in management posts. This hierarchy is shared by the newly incorporated nurses. Nurses are the ones in charge of receiving, training and preparing the new nurses relocated to primary care. Furthermore, they consider that they should be allowed to participate in decision making, regarding the policies and management of the incorporation of new nurses. Nurses assume that a value of the nursing profession is that individualized training exists from the more veteran nurse to the novice nurse. For nurses from the health clinic, the basis of relationships with other professionals is the existence of a relationship of reciprocity, and teamwork, without hierarchies among members of the health clinic care team, where the nurse can lead and coordinate the health clinic, and the different health programs offered.

**Table 3:** Question guide.

Investigated Theme	Questions
Staff policies applied.	How do you believe human resource policies affect the way care is performed? And in primary care? How is leadership from your institution in primary care? How is it in nursing? How do you believe that this leadership must be? From the outside to the inside, from the outside to the teams, or from the inside out, or should the members of the team itself be the ones to take leadership? What barriers and facilitators exist for leadership on behalf of primary care nurses?
Beliefs regarding the newly incorporated nursing staff	What are the strengths of the PC nurse and what are the weaknesses? What opinions do you think there are regarding the PC nurse compared to the hospital nurse? Are there many differences between the work of some nurses compared to others? Are there important differences between the first generation and the next generations? What do newly incorporated nurses add to current work dynamics? Highlight positive and negative aspects.
Reasons for relocation to primary care	What were the reasons that drove the relocation from the hospital to primary care? Does primary care meet your expectations? Do you consider that you were sufficiently qualified in primary care when you arrived from the hospital? What barriers and facilitators can you highlight for transferring form the hospital to primary care?
Concern for the future	How do you currently perceive primary care since you arrived? What changes have been the most relevant for you? How has this affected primary care? And nursing? And community care?

**Table 4:** Trustworthiness criteria.

Criteria	Techniques Performed and Application Procedures
Credibility	Investigator triangulation: each interview, observation and researcher note was analyzed by two researchers. Thereafter, team meetings were performed in which the analyses were compared and themes were identified. Participant triangulation: the study included participants with different characteristics. Triangulation of data collection methods: unstructured interviews, semi-structured interviews, non-participant observations were conducted and researcher field notes were kept. Participant validation: this consisted of asking the participants to confirm the data obtained during the stages of data collection and analysis. All participants were offered the opportunity to review the audio or written records as well as the subsequent analysis to confirm the interpretation of their narratives by the researchers.
Transferability	In-depth descriptions of the study performed, providing details of the characteristics of researchers, participants, contexts, sampling strategies, and the data collection and analysis procedures.
Dependability	Audit by an external researcher: an external researcher assessed the study research protocol, focusing on aspects concerning the methods applied and the study design. Also, an external researcher specifically checked the description of the coding tree, the major themes, the participants’ quotations, the identification of quotations, and the descriptions of themes.
Confirmability	Investigator triangulation, participant triangulation, and data collection triangulation. Researcher reflexivity was encouraged via the performance of reflexive reports and by describing the rationale behind the study.

Credibility: Confidence in the truth of the findings; Transferability: reporting that the findings have applicability in other contexts; Dependability: reporting that the findings are consistent and could be repeated; Confirmability: the degree to which findings were determined by the respondents and not by the biases, motivations, or interests of researchers [7,31,32].

**Table 5:** Literal excerpts from the interviews.

**Theme 1. Staff policies applied.**
*“From my point of view, according to what I have experienced, people who come from the hospital have left mainly because at hospital they were experiencing burnout and they go to the clinics with an expectation that the work will change, a different timetable, without shifts, but I think that they also think that it is going to be a more “cushy” job.”* E04
*“I think that there have also been disastrous relocation strategies to clinics, such as those who have relocated automatically, because of seniority, people who have relocated directly without any previous preparation, without any knowledge whatsoever, going from hospitals to primary care.”* E03
*“In our profession, relocations are performed in a way that favors people getting out of the hospital.”* E03
*“When there have been staff changes, we have really noticed this and we have experienced many conflicts. This has always coincided with these types of relocations.”* E03
**Theme 2. Beliefs regarding the newly incorporated nursing staff.**
*“When there were relocations, a colleague from Valdecilla came who was also quite older. We met this with fear. We feared that, somehow, this was a deterrent for the rest of the team, in the sense that you think: Now the old glories of Valdecilla come to the elephants’ graveyard.”* E12
*“People from hospital thought that they came to primary care in order to work less (…). They had no idea (…).”* E02
*“… it is the image that I think people have of primary care, that you come here to rest and relax, you leave the hospital….* “ A02
*“There are people who want to come because they consider it to be an interesting change in terms of shifts or whatever, and there are people who believe that here you take people’s blood pressure four times and then you can sit back and wait for retirement.”* E07
*“In hospital you are working more or less on the side, in a specialized environment, with a patient who goes a certain number of days, who has an exacerbation, or whatever, then he goes and it’s over. But of course, here you are in the patient’s life, you go to their home and at times you have no idea how to manage the situation. They can ask you things, conflicts, stressful situations arise, which you don’t know how to manage.”* E03
*“Besides, I have a colleague who is a hard worker. She comes from the hospital, where she has worked forty years and she is a very hardworking person with a lot of will to learn, but she has a totally different conception of nursing compared to ours. I see her as being different.”* E05
**Theme 3. Reason for relocation to primary care**
*“All the noise was getting to my head in the hospital environment. There were too many people, too many things, too much noise. I needed a bit of silence and then, all of sudden, not only did I want to go to a health clinic, but also I wanted to go to a village.”* E05
*“I went to primary care because I only had a few years left to go before I retired and I wanted to leave my profession with a good feeling. I needed some kind of stimulus, I needed to find the career I had started with so much hope; I didn’t want my life to end like that. I was quite disenchanted with my profession, I was very tired, the circumstances in which I was working didn’t seem like the most appropriate, the work I was doing did not seem enriching.”* E10
*“I feel like getting to know primary care and also, it’s good in order to not continue working each month one week in the morning, another in the afternoon, and then at night.”* A01
**Theme 4. Concern for the future.**
*“Half of the team goes and the other doesn’t want to… Look, it is quite unusual because us four young nurses who are there, all of us are leaving. I think that if we continued in that team, because the team is established and formed, and because we arrived beforehand over the “last years, now is when we could begin to do something ourselves.”* E05
*What’s going on? The fact that people like yourself, many people have gone to a last post, people who were already doing lots of things, and who still had much to do.”* E07
*“They are changing the team a lot, Young people come, but they change us around. What problem does this bring? It means you cannot become too involved.”* E06
*“The fact that one day, someone arrives to cover for another person, and the next day someone else comes, is dreadful… there is no continuity of care.”* E31

## Supplementary Materials

The following are available online at https://www.mdpi.com/1660-4601/16/19/3653/s1, Table S1. Coreq Checklist
